# Targeting and microenvironment-improving of phenylboronic acid-decorated soy protein nanoparticles with different sizes to tumor

**DOI:** 10.7150/thno.33470

**Published:** 2019-10-11

**Authors:** Xiaoping Qian, Lei Ge, Kangjun Yuan, Cheng Li, Xu Zhen, Weibo Cai, Rongshi Cheng, Xiqun Jiang

**Affiliations:** 1 MOE Key Laboratory of High Performance Polymer Materials and Technology, Department of Polymer Science & Engineering, College of Chemistry & Chemical Engineering, and Jiangsu Key Laboratory for Nanotechnology, Nanjing University, Nanjing, 210023, People's Republic of China; 2 Department of Radiology, University of Wisconsin-Madison, Madison, WI 53705;

**Keywords:** Soy protein, phenylboronic acid, size effect, tumor microenvironment, drug delivery.

## Abstract

It is essential for nanoparticles to delivery drugs accurately and penetrate deeply to tumor. However, complicated tumor microenvironment such as elevated tumor interstitial fluid pressure (IFP) and solid stress reduces the transport efficiency of nanomedicines in tumor.

**Methods:** We herein report a drug delivery system of phenylboronic acid-decorated soy protein nanoparticles with the size of 30 nm, 50 nm and 150 nm. *In vitro* examinations including cytotoxicity, cellular uptake and penetration in multicellular tumor spheroids and *in vivo* observations including IFP and tumor solid stress measurements and antitumor activity were performed.

**Results:** It was found that phenylboronic acid moiety could endow the nanoparticles actively targeting affinity to sialic acid (SA) which overexpressed in tumor cells. Simultaneously soy protein could improve tumor microenvironment such as reduction of IFP and tumor stress. Among the soy protein nanoparticles with different sizes, 30 nm-sized nanoparticles showed the best cellular uptake and highest cytotoxicity *in vitro* after loading doxorubicin (DOX). *In vivo*, 30 nm-sized nanoparticles showed the best tumor microenvironment improvement efficiency, leading to the enhanced drug accumulation and antitumor efficiency when combination with DOX.

**Conclusion:** Our study introduces a bioactive nanoparticulate design strategy to actively target and significantly improve tumor microenvironment for enhanced cancer therapy.

## Introduction

Nanomedicines play a vital role in tumor treatment since they can improve therapeutic efficacy and lower side effects to a great degree by transporting therapeutic drug to tumor site in active or passive mode 1. Among these nanomedicines, polymer-based nanoparticles are extremely attractive due to their excellent stability, biocompatibility and biodegradation 2-6. However, there are still many obstacles for nanoparticles to enter into tumors due to the complex tumor microenvironment.

Solid tumors are composed of tumor cells with extracellular matrix 7. To effectively transport antitumor drugs to desired sites, the drug delivery systems should go through a process of circulation, accumulation, penetration, internalization and drug release 8. However, such a progress is challenged by the complicated tumor microenvironment, such as collapsed tumor vessels, elevated solid stress, dense extracellular matrix and high tumor interstitial fluid pressure (IFP) 9. A lot of efforts have been made to improve tumor penetration through decreasing tumor IFP. For example, angiotensin inhibitor losartan could decrease tumor IFP and increase vascular perfusion. The decrease of tumor IFP by losartan was associated with reduction of stromal collagen and hyaluronan production, a decrease in the expression of profibrotic signals transforming growth factor (TGF)-β1, connective tissue growth factor (CCN2) and endothelin-1 (ET-1) 10. The size of the nanoparticles is another important factor affecting the tumor penetration 11-13. Thus, it is highly desirable to develop a bioactive nanomedicine with suitable size which can penetrate deeply in tumor sites by improving tumor microenvironment to get a better drug delivery efficiency.

Soy protein (SP) is a natural plant protein extracted from soy, and has been widely used as biomedical materials for many years 14-16. It is mainly made up of glycinin (40%) and β-conglycinin (30%). These two components determine the basics nature of SP 17. Glycinin has five different subunits. Each subunit is made up of an acidic polypeptide (about 35 k Da) and a basic polypeptide (about 20 k Da) and they are bound with a disulfide 18, 19. SP contains all of the essential amino acids and also offers a good balance among nonpolar amino acid, polar amino acid and charged amino acid. SP has been investigated as the food additives, water emulsions and nutraceutical encapsulation in the form of nanoparticles, microspheres and hydrogels 20-23. It was reported that the hydrolysate of SP had the ability to inhibit angiotensin I-converting enzyme (ACE) which could be used to treat hypertension 24 and the ACE inhibition peptides derived from SP could lower blood pressure of spontaneously hypertensive rats 25.

**Scheme 1:**
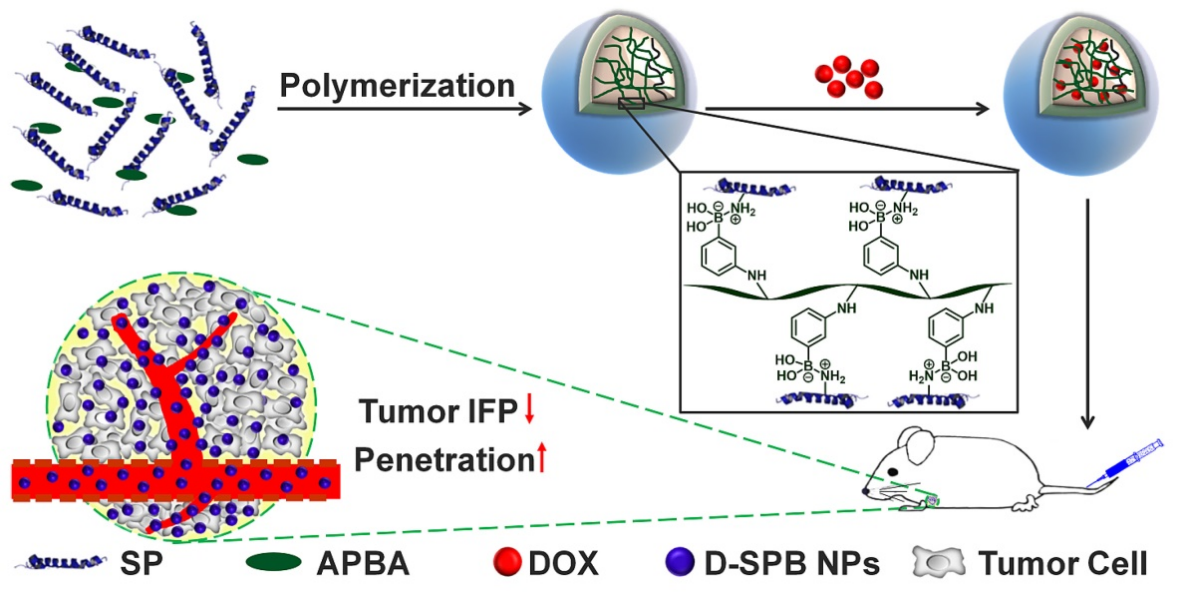
Schematic illustration of synthesis and delivery of SPB NPs *in vivo*.

With these in mind, we hypothesized that they might also decrease tumor IFP and tumor solid stress. In this study, we report the design of phenylboronic acid-decorated soy protein nanoparticles (SPB NPs) that can actively target and improve tumor microenvironment for enhanced cancer therapy simultaneously (**Scheme 1**). The nanoparticles have two advantages. One is that the phenylboronic acid group in the SPB NPs can actively bind to the sialic acid (SA) residues overexpressed on the surface of tumor cells 5, 26-30. The other advantage is that SP could decrease tumor IFP and solid stress for normalizing tumor microenvironment. Such a unique dual-functional feature of SPB NPs results in the greatly simplified nanomedicine system with enhanced cancer therapy. In the following, we first described the synthesis and characterization of SPB NPs, followed by *in vitro* and *in vivo* validation of SPB NPs' ability to actively target the SA overexpressed tumor cells and improve tumor microenvironment such as reduction of tumor IFP and solid stress. At last, the proof-of-concept application of the SPB NPs combination with DOX in the treatment of SA overexpressed tumor in living mice is demonstrated.

## Materials and Methods

### Materials

SP was bought from Shanghai Yiji Biotech Co., Ltd. (Shanghai, China). N-3-aminophenylboronicacid (PBA) was bought from Beihua Xinyuan Science & Technology Developing Co., Ltd. (Beijing, China). Acryloyl chloride (AC) was bought from J&K Scientific. 4,4'-Azobis(4-cyanovaleric acid) (ACVA) and FITC isothiocyanate were purchased from Sigma Chemical Co. Doxorubicin (DOX) was purchased from Shenzhen Main Luck Pharmaceuticals Inc. (Shenzhen China). 3-(4,5-Dimethylthiazol-2-yl)-2,5-diphenyltetrazolium bromide (MTT) was purchased from BDH Laboratory Supplies (Poole, Dorset, England). Hoechst 33342, 4',6-diamidino-2-phenylindole (DAPI), Dulbecco's modified eagle medium (DMEM) and Roswell park memorial institute (RPMI)-1640 were purchased from Jiangsu KeyGEN BioTECH Corp., Ltd. Rat anti-mouse CD31 was obtained from BD Pharmingen. Donkey anti-rat Alexa-594 was obtained from Invitrogen. Anti-collagen I antibody, anti-hyaluronic acid antibody, anti-TGF beta 1 antibody and goat anti-rabbit IgG H&L (Alexa Fluor 488) were obtained from Abcam. All other reagents were of analytical grade and were used without further purification.

Human neuroblastoma tumor cell (SH-SY5Y), murine hepatic tumor cell (H22), human liver tumor cell line (HepG2) and mouse colon carcinoma cell (CT26) were obtained from Shanghai Institute of Cell Biology (Shanghai, China). Male ICR and BALB/c mice (4-6 weeks old, weighing 18-20g) were provided by Animal Center of Drum Tower Hospital (Nanjing, China). All animal studies were performed in compliance with guidelines set by the Animal Care Committee at Drum-Tower Hospital.

### Purification of SP

One gram of SP was put into 50 mL distilled water and the pH value was adjusted to 6.5. After stirring for 30 min at room temperature, the solution was centrifuged at a speed of 5,000 rpm to get rid of any insoluble contamination. The supernatant was then dialyzed for two days against distillated water and finally lyophilized. The molecular weight and purity of SP were detected by sodium dodecyl sulfate polyacrylamide gel electrophoresis (SDS-PAGE).

### Preparation of SPB NPs

SPB NPs were prepared based on a polymer-monomer pair reaction system 31. First, N-3-acrylamidophenylboronic acid (APBA) was prepared by the reaction of PBA with AC based on our previous study 32. Then, 50 mg purified SP and 50 mg APBA were dissolved in 10 mL of deionized water. 10 mg initiator ACVA was added into the reaction system after the solution became clear and transparent. After the pH value of the reaction system changed to 5.5, the polymerization of APBA monomer was initiated in the presence of SP at 80^ o^C under argon atmosphere. A light blue suspension appeared slowly and the reaction was proceeded for another 0.5 h. The suspension was cooled to room temperature and the large aggregates were removed through filtration. The resulting suspension was dialyzed against distilled water for 48 h using a dialysis bag (MWCO 100 kDa) and three sizes of SPB NPs (30 nm (SPB30), 50 nm (SPB50), 150 nm (SPB150)) were separately obtained by changing the pH value of the initial solution.

### Characterization of SPB NPs

The mean diameter and size distribution of SPB NPs in aqueous solution and the stability of SPB NPs in deionized H_2_O, PBS (0.01 M, pH 7.4), saline, DMEM and 10% FBS were analyzed by dynamic light scattering (DLS) (Brookheaven Instruments Corporation, USA). All measurements were conducted at 25 ^o^C with a laser wavelength of 660.0 nm. Zeta potential was determined by Zetaplus and the morphology was observed by transmission electron microscopy (TEM, JEM-1011, Japan).

### Preparation of DOX-loaded SPB (D-SPB) NPs

D-SPB NPs were obtained by adding an aqueous solution of DOX into SPB NPs at a pH value of 8.0. It was then stirred overnight in the dark. Free DOX was removed by ultrafiltration and DOX-loaded SPB30 (D-SPB30), DOX-loaded SPB50 (D-SPB50) and DOX-loaded SPB150 (D-SPB150) were obtained. The drug loading content (DLC) of D-SPB NPs was determined by a UV-vis spectrometry (UV-5300, Shanghai Metash Instruments Co., Ltd, China). Standard DOX solution with different concentrations was used to make a calibration curve under the same conditions. The DLC and drug loading efficiency (DLE) were determined by the formulas mentioned below:

DLC (%) = (Mass of DOX in SPB NPs) / (Mass of the D-SPB NPs) × 100%

DLE (%) = (Mass of DOX in SPB NPs) / (Mass of feeding DOX) × 100%

### Drug release from D-SPB NPs *in vitro*

1 mL aqueous solution of D-SPB NPs or free DOX (with the same concentration of DOX) was enclosed with a dialysis bag (MWCO, 14 kDa). It was then placed into PBS (0.01 M, 5 mL). The pH value of PBS was chosen as 5.0, 6.0 and 7.4, respectively. The solution was slowly shaken at 37^ o^C in the dark. The released medium was withdrawn at some definite time points and fresh PBS (0.01 M, 5 mL) was added. The amount of DOX in released medium at different time was measured by a UV-vis spectrophotometer. Accumulative release amount of DOX was plotted versus time and calculated.

### Cellular Uptake of SPB NPs *in vitro*

To systematically estimate the cellular uptake ability of SPB NPs, SH-SY5Y cell lines (normal SA expression), H22 cell lines (1.8-fold SA expression 28) and HepG2 cell lines (6-fold SA expression 33) were used. FITC was used to label SPB NPs. Briefly, 10 mg SPB NPs was dissolved in distilled water. 2 mg FITC dissolved in 1.0 mL DMSO was added at pH value of 8, followed by stirring overnight in the dark at room temperature. Then, free FITC was removed by ultrafiltration several times and FITC-labeled SPB NPs were obtained. The cells were seeded at a density of 1 × 10^5^ per well in a six-well plate with a cover glass for 24 h, and then the same concentration of FITC-labeled SPB NPs were added. After co-incubation for 4 h at 37 ^o^C, cells were washed three times with sterile PBS and fixed with 4% paraformaldehyde. The final cellular uptake ability of SPB NPs was examined by confocal laser scanning microscopy (CLSM, Zeiss LSM 710, Germany). Flow cytometry was used to quantitatively calculate the amount of cellular uptake. Briefly, a density of 1 × 10^5^ cells was seeded in 24-well plates for 24 h. FITC-labeled SPB NPs were added and co-cultured for 4 h at 37 ^o^C. Cells were digested, centrifuged, washed with sterile PBS for three times and detected by flow cytometry (BD Accuri C6).

To study to location of SPB NPs after cellular uptake, HepG2 cells were seeded with a density of 1 x 10^5^ per well for 24 h. The cells were co-cultured with Lyso Tracker (red) for 30 min at 37 ^o^C 34. The addition of FITC-labeled SPB NPs and subsequent processing were the same as described above. To study the active targeting properties of SPB NPs, HepG2 was pretreated with APBA for 1 h before adding FITC-labeled SPB30. Cell culture and other operations were the same as described above.

### Penetration of SPB NPs in multicellular tumor spheroids (MCTs)

The three-dimensional (3-D) CT26 and HepG2 MCTs were prepared as previously described 26. When the diameter of MCTs became larger than 200 μm, 5 mL Eppendorf tubes were used to collect about 25 spheroids each. FITC-labeled SPB NPs were added and co-cultured for 24 h at 37 ^o^C. The MCTs were washed with sterile PBS before detected by CLSM.

### Cytotoxicity of D-SPB NPs *in vitro*

Cell cytotoxicity D-SPB NPs *in vitro* against SH-SY5Y, H22 and HepG2 cells were evaluated by MTT assay, blank SPB NPs and free DOX were used as control groups. Cells were placed on 96-well plates with 5,000 cells per well and cultured in the corresponding medium at 37^ o^C for 24 h. Different concentrations of SPB30, SPB50, SPB150, free DOX, D-SPB30, D-SPB50 and D-SPB150 were co-incubated with the cells for another 24 h. The medium was withdrawn and 200 μL fresh culture medium containing 20 μL MTT (5 mg/mL in sterile PBS solution) was added. The medium was withdrawn again after 4 h and 150 μL DMSO was added with gently oscillate to dissolve the formazan. The absorbance of each well was detected at 570 nm by a microplate reader (Huadong, DG-5031, NJ).

### Measurement of tumor IFP and solid stress

ICR mice were planted with H22 cells subcutaneously at the left flank. When the tumor grew up to about 1 cm^3^, SPB NPs were intravenously (i.v.) injected through the tail vein. The tumor IFP was measured at specific time points by multi-channel physiological signal acquisition system (Chengdu Instrument Factory, China). Every tumor was measured more than three times. Hoechst 33342 was i.v. injected 24 h after injection of SPB NPs and the mice were sacrificed five minutes later. A total of 5 mL saline was then injected through the left atrium to push out the blood from mice. Finally, the tumors were collected, cut into 20 μm sections after frozen and treated for CLSM observation. BALB/c mice were planted with CT26 cells subcutaneously at the left flank. When the tumor grew up to about 1 cm^3^, a dose of 150 mg/kg of SPB NPs was i.v. injected through tail vein. The mice were sacrificed humanely after 24 h and tumors were excised. The collected tumors were cut through the middle to about 80% along the longest diameter and put in saline for 5 min. The tumor opening was measured and solid stress was calculated 35 as a function of particle size. To explore the mechanism of tumor IFP and solid stress reduction, H22 tumors before and after treatment of SPB NPs were cut into sections with a thickness of 20 μm and stained with antibodies of collagen I, hyaluronan and TGF-β1. Alexa 488 (goat anti-rabbit) was chosen as the secondary antibody to detect collagen, hyaluronan and TGF-β1.

### Tumor penetration of SPB NPs *in vivo*

To determine the penetration ability of SPB NPs *in vivo*, FITC-labeled SPB NPs were i.v. injected into H22 tumor-bearing mice. After 24 h administration, the mice were sacrificed and tumors were collected in 4% paraformaldehyde aqueous solution at 4 ^o^C. The tumors were then dehydrated with 30% sucrose solution for 12 h and cut into 9 μm sections after frozen in optimal cutting temperature for histological analysis. To stain tumor slices, the tumor sections were rehydrated in PBS, followed by incubation with 3% BSA aqueous solution. The tumor sections were dyed with monoclonal antibody CD31 for 1 h and washed three times with PBS (pH 7.4 with 0.05% (v/v) tween 20). The secondary antibody (Alexa 594 conjugated donkey anti-rat antibody) were used to stain the tumor vessels and the tumor sections were dyed with DAPI to mark the nuclei. CLSM was used to observe the resulting tumor sections after mounted with mounting medium.

### Biodistribution of DOX in H22 tumor-bearing mice

The *in vivo* biodistribution of DOX was examined according to our previous work 26, 28, 36. ICR mice were planted with H22 tumor cells subcutaneously at the left flank. When the tumors grew up to about 500 mm^3^, mice were randomly divided into two groups. One group was injected with SPB30 at a dosage of 150 mg/kg every day for three days, then free DOX (4 mg/kg) was i.v. injected together with another group. The blood was collected and the mice were sacrificed at several predetermined time after administration (N = 3 at each time point per group). Subsequently, major organs such as heart, liver, spleen, lung, kidney and tumor were excised and weighed. The blood was centrifuged at a speed of 14,000 rpm to get the plasma. The plasma and major organs were suspended in 70% ethanol with 0.3 M HCl, intensely homogenized and put in the dark for 48 h. A further centrifugation was done and the fluorescence intensity of DOX in the supernatants were detected by a fluorescence spectrometer at an excitation wavelength of 480 nm and emission wavelength of 590 nm. A standard curve of DOX was made and the DOX concentration in each sample was calculated by this standard curve. Three mice treated with PBS were used as a control group. The tissue samples from control group were served as background. To study the biodistribution of DOX from different sizes of D-SPB NPs, the H22 tumor-bearing mice were i.v. injected with free DOX, D-SPB30, D-SPB50 and D-SPB150 at a dose of 4 mg/kg for DOX concentration, respectively. The mice were sacrificed and the blood together with major organs were excised and weighed just like the process described above.

### Antitumor efficiency of SPB NPs

ICR mice were planted with H22 tumor cells subcutaneously at the left flank when the body weight reached to 20-22 g per mouse. When the tumor volume reached to about 50 mm^3^, ten mice were pretreated with 150 mg/kg SPB30 every day for three days. Then, free DOX (4 mg/kg) was i.v. injected together with another ten mice and this day was marked as day one. The longest and shortest diameter (a and b) of tumor was surveyed by a Vernier caliper every other day and tumor volume (V) was figured out as V = a × b^2^/2. The tumor growth inhibition (TGI) was calculated as TGI = (1-volume of experimental group/volume of control group) × 100 %. The body weight of mice was loaded every two days and the survival numbers of mice were counted until the end of this experiment. To study the antitumor efficiency of different sizes of D-SPB NPs, H22 tumor-bearing mice were randomly divided to eight groups with 10 mice in each group. Saline, SPB30, SPB50, SPB150, free DOX (4 mg/kg), D-SPB30 (4 mg/kg DOX eq.), D-SPB50 (4 mg/kg DOX eq.) and D-SPB150 (4 mg/kg DOX eq.) were i.v. injected into the mice, respectively, and this day was marked as day one. The antitumor efficiency was evaluated just like the process described above.

### Statistical analysis

Error bars are reported as means ± S.D. Analysis of variance (ANOVA) was employed to compare the difference of data and P value less than 0.05 was considered statistically significant.

## Results and discussion

### Preparation and characterization of SPB NPs

The molecular weight and purity of SP pretreated were examined by SDS-PAGE and an acceptable purity of water-soluble SP with a molecular weight of about 35 kDa was obtained (**Figure S1**). The molecular weight was comparable to the acidic polypeptide in the subunit of glycinin. Subsequently, phenylboronic acid-contained SPB NPs were prepared through a polymer-monomer pair reaction system made up of a water-soluble SP and a polymerizable monomer APBA. Briefly, APBA was polymerized in the presence of SP in aqueous solution by radical initiating reaction. Along with the polymerization of APBA, the boronic acid groups of poly(N-3-acrylamidophenylboronic acid) interacted with amino and guanidyl groups in SP, resulting in the nanoparticle formation 28. ^1^H NMR characterization of free SP, free PAPBA and SPB NPs confirmed that both SP and PAPBA were in SPB NPs. The characteristic peaks of SP were mainly located at 0.7-0.9 ppm, 3.7-4.5 ppm, while the characteristic peaks of PAPBA were mainly located at 6.9-7.4 ppm. All of the characteristic peaks of SP and PAPBA could be found for SPB30, SPB50 and SPB150 (**Figure S2**). This result verifies the successful synthesis of phenylboronic acid-decorated SPB NPs.

**Figure 1:**
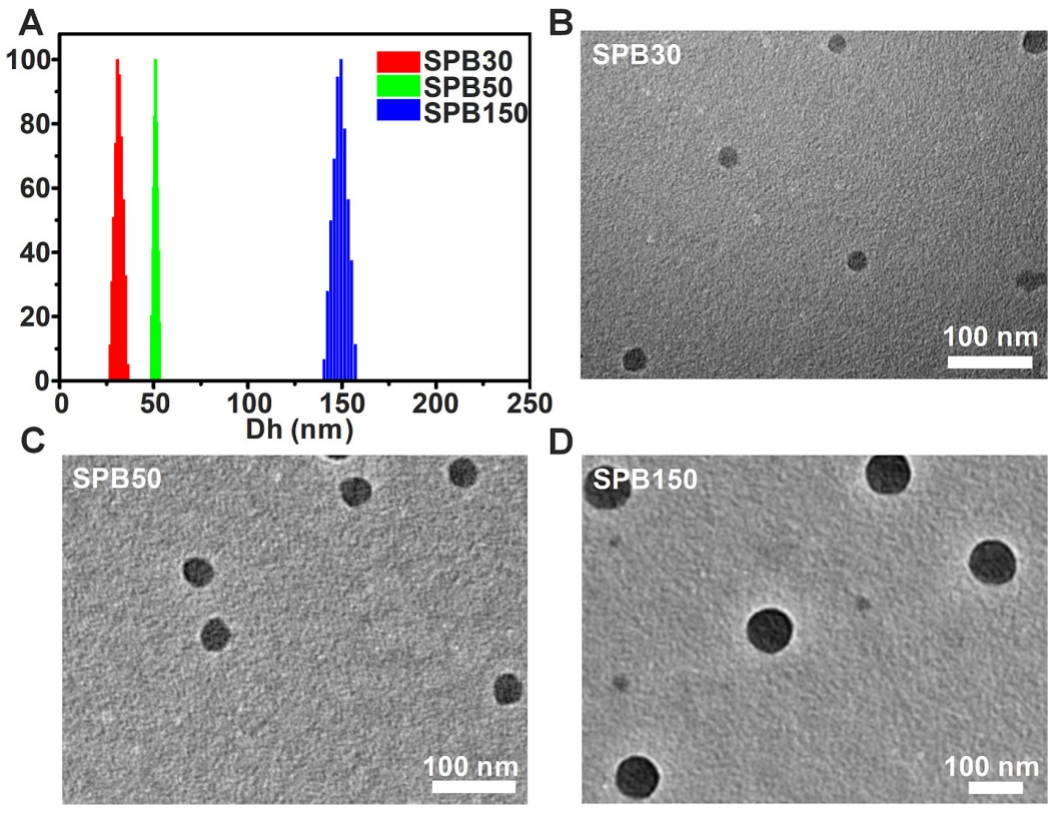
** Size and morphology of SPB NPs.** The hydrodynamic diameter distribution of SPB30, SPB50 and SPB150 (A); TEM images of SPB30 (B), SPB50 (C) and SPB150 (D).

The D_h_ of SPB NPs were measured by DLS and TEM, respectively. All samples displayed a unimodal size distribution in DLS measurement (**Figure 1**). Under different preparation condition, the hydrodynamic diameter of SPB NPs was 30 nm, 50 nm and 150 nm, respectively, as shown in **Table 1** and **Figure 1A**. No obvious change in diameters of SPB NPs were found in H_2_O, PBS, saline, DMEM and 10% FBS during 96 hours, suggesting their high stability in different media (**Figure S3**). TEM observation showed that SPB30, SPB50 and SPB150 samples had well uniform spherical shapes (**Figure 1B, C and D**).

### DOX loading and *in vitro* release of SPB NPs

The DOX loading ability of SPB NPs was influenced mostly by the feeding ratio of nanoparticles to DOX and a drug loading content up to 27% could be obtained by changing the feeding dosages of DOX. Meanwhile, the encapsulation efficiency of DOX was larger than 90%, suggesting the great ability of SPB NPs for loading DOX (**Table 2**). The high drug loading content might be attributed to the intense coordination interaction between the amino group in DOX and the electron-deficient boron in SPB NPs. In subsequent experiments, a DOX loading content of 25% was used unless additional stated. DOX-loaded SPB NPs were named as D-SPB NPs. The DOX release profiles from D-SPB NPs were evaluated in PBS (0.01 M) with pH 5.0, 6.0 and 7.4, respectively, and the solution was placed into a dialysis bag. The free DOX in dialysis bag diffused rapidly and about 100% of free DOX could spread out of dialysis bag within 5 hours (**Figure S4A**). This ensures the measurement accuracy for DOX release from D-SPB NPs. The DOX release process from D-SPB NPs was pH dependent for all the nanoparticles and more DOX was released from the nanoparticles as pH decreased (**Figure S4B-D**). For D-SPB30, the released DOX was limited to 26% until 120 h at pH 7.4, while the cumulative release increased to 39% at pH 6.0 and 77% at pH 5.0, respectively. The D-SPB50 and D-SPB150 showed a similar release trend to D-SPB30. This pH-dependent DOX release behavior might ascribe to two reasons. One was due to the weaker coordinate interaction between the electron-donating amino group of DOX and the electron-deficient boron of APBA when the pH value decreased. Another was the increased water-solubility of DOX as pH of medium dropped. This release behavior dependent on pH was necessary to controlled drug release in tumor since there would be little drug released in the systemic circulation (neutral environment) and more in the tumor tissue and intracellular sites (acidic environment) 37, 38.

### Cellular uptake *in vitro*

**Figure 2:**
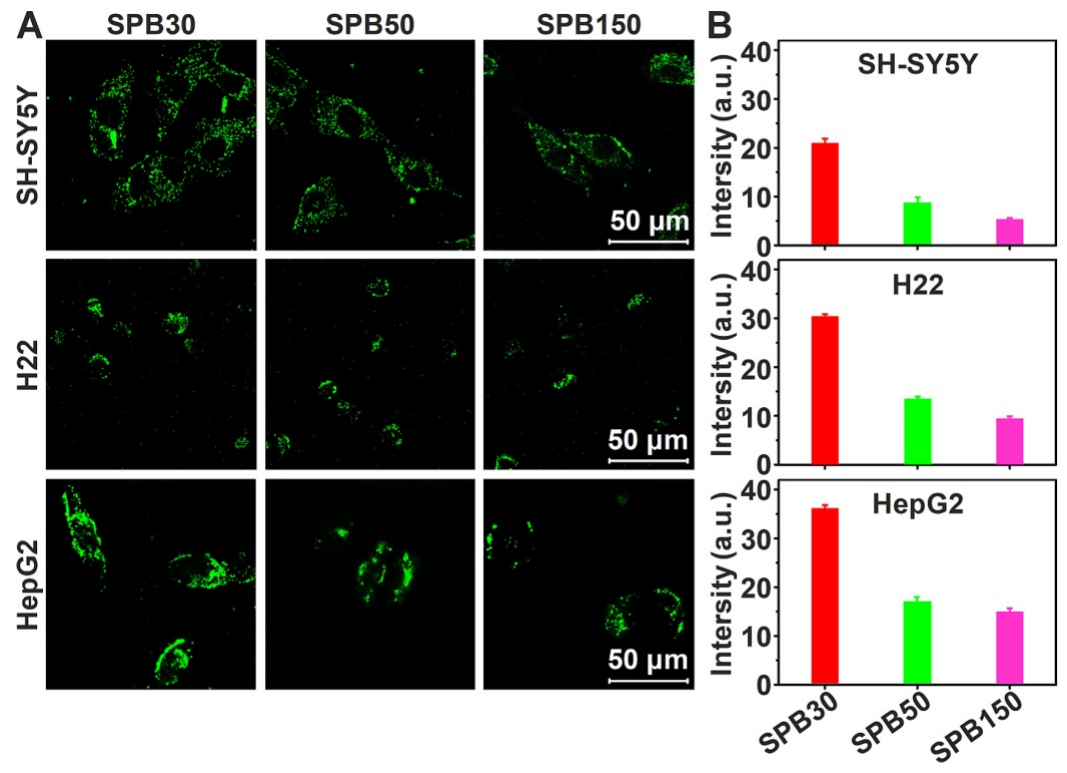
** Cellular uptake of SPB NPs.** CLSM images of SH-SY5Y, H22 and HepG2 cells incubated with FITC-labeled SPB NPs (A); Quantitative results for the relative fluorescent intensity of SPB NPs in cellular region determined by flow cytometry (B). Data is represented as mean ± SD (N = 3).

It was reported that the expression of SA on H22 tumor cells was 1.8-1.9 times that of hepatocytes 28 and the expression of SA on HepG2 tumor cells was 6 times that of normal liver tissue cells 33. To observe the cellular uptake ability of SPB NPs by the cells with different expression of SA, FITC-labeled SPB NPs were incubated with SH-SY5Y (SA-negative cells), H22 (SA-overexpressed cells) and HepG2 (SA-overexpressed cells) cell lines at 37 ^o^C for 4 h and then observed by CLSM. SPB30 displayed a higher fluorescence than SPB50 and SPB150 in all the cell lines. All the nanoparticles showed a higher fluorescence in HepG2 cells, compared to that in H22 and SH-SY5Y (**Figure 2A**). Furthermore, the fluorescence intensity of FITC was quantitatively analyzed by flow cytometry. The fluorescence intensity of SH-SY5Y, H22 and HepG2 cells incubated with SPB30 was 3.90, 3.20 and 2.44 folds higher than that of SPB150, and 2.36, 2.26 and 2.10 folds higher than of SPB50 (**Figure 2B**). The fluorescence intensity of SPB30 in HepG2 was higher than H22 and SH-SY5Y. These results suggest that SPB NPs can preferentially internalized by SA-overexpressed cells, and the smallest SPB30 have the greatest cellular uptake ability in the SA-overexpressed tumor cells.

To observe the distribution of SPB NPs in the cells *in vitro*, HepG2 cells were pretreated with Lyso Tracker (red) for 1 h to mark the endosomal and lysosomal. Then, FITC-labeled SPB NPs were co-cultured with the pretreated cells for another 4 h before observed by CLSM. In the cytoplasm region, strong green fluorescence punctate patterns were observed (**Figure S5A**). The nanoparticles distributed mostly in endosome and lysosomes, as indicated by a yellow sight merged by red (endosomal/lysosomal) and green (SPB NPs). Besides, no fluorescence was found inside nucleus region, suggesting that all of the SPB NPs are unable to penetrate through the cell nuclear membrane (**Figure S5A**). These results indicate that SPB NPs enter the cells mainly with an endocytosis manner. To study the necessity of APBA moiety in the nanoparticles, a competition test was performed. HepG2 cells were pretreated with APBA for 1 h before incubating with FITC-labeled SPB30. Compared to the pretreated group, more SPB30 NPs were taken up by HepG2 cells without treatment (**Figure S5B**). According to the cell flow cytometry, the amount of SPB30 taken up by HepG2 cells without treatment was 1.43 times that of the group with APBA pretreatment (**Figure S5C**). Thus, it can be concluded that APBA moiety in SPB NPs can bind to SA and plays a great role in cellular uptake of SA overexpressed cells.

### Penetration of SPB NPs in MCTs

**Figure 3:**
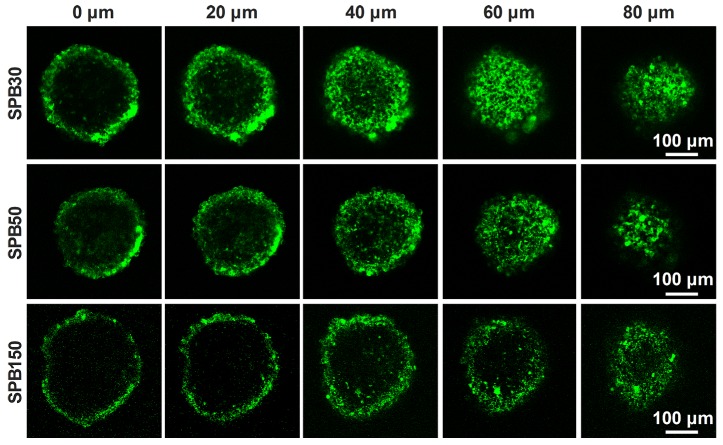
Representative Z-stack CLSM images of CT-26 MCTs incubated with FITC-labeled SPB NPs for 24 h were obtained starting in the middle of the spheroid in 20 μm intervals.

Penetration of nanoparticles in deep tumor site is very important for receiving a desirable therapeutic efficacy. MCTs were used to investigate the effect of particle size on the penetration ability of SPB NPs in 3-D cells *in vitro* due to the closely cell packing and dense extracellular matrix. In order to eliminate the effect of SA on the penetration, the SA-negative CT26 cell model was selected to construct 3-D multicellular tumor spheroids for simulating tumor tissue. As shown in **Figure 3**, although all sizes of SPB NPs could enter into CT26 MCTs, most of them were located in the vicinity of periphery of CT26 MCTs. Among the nanoparticles with different sizes, SPB30 showed the strongest fluorescence signal and deepest penetration in CT26 MCTs, indicating a size-dependent penetration of SPB NPs in CT26 MCTs. The 3-D multicellular tumor spheroids of SA-overexpressed HepG2 cells were also used to study the effect of SA on penetration of SPB NPs in MCTs. As shown in Figure S6, there was no obvious difference in penetration in MCTs between CT26 and HepG2 MCTs, indicating that the expression level of SA had little effect on penetration of SPB NPs in MCTs. On one hand, SA-positive HepG2 cells facilitates cellular uptake of SPB NPs in 3D MCTs. On the other hand, high SA expression in HepG2 MCTs reduces for deeper penetration of SPB NPs in MCTs, leading to binding site barrier. Thus, compared to SA-negative CT26 MCTs, SA-positive HepG2 MCTs have not display significant merit in the penetration of SPB NPs.

### Cytotoxicity *in vitro*

**Figure 4:**
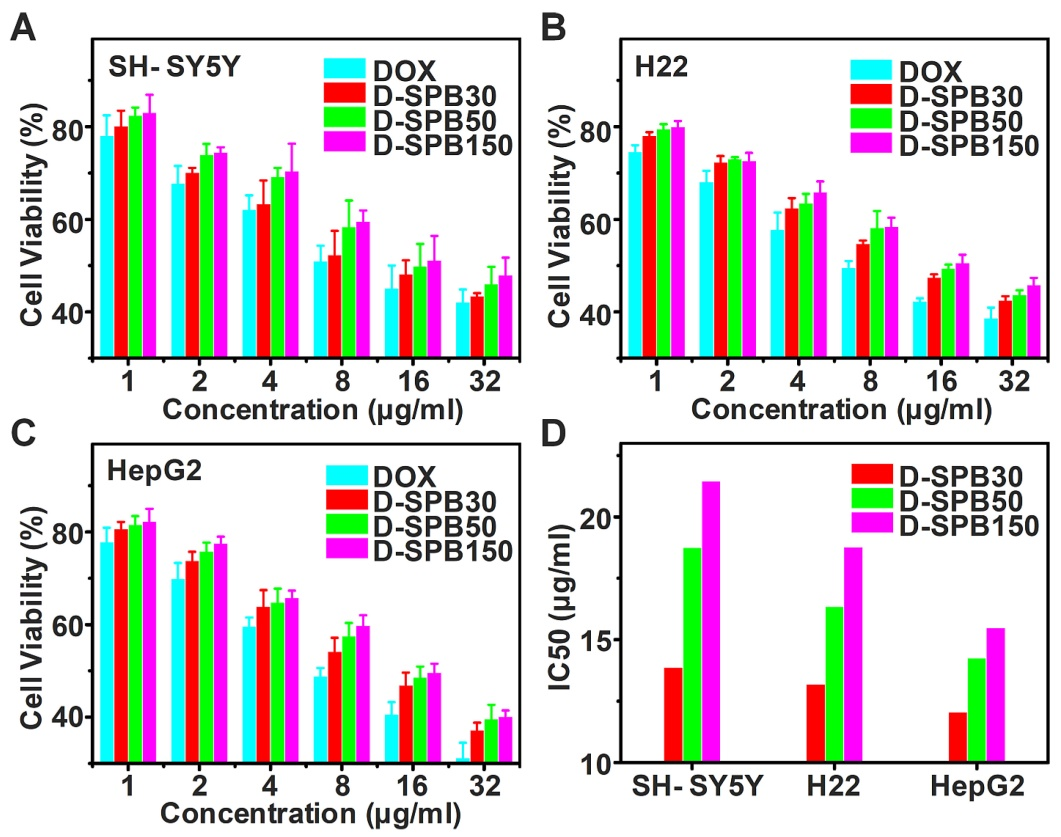
** Cytotoxicity of D-SPB NPs *in vitro*.** *In vitro* cytotoxicity of D-SPB NPs against SH-SY5Y (A), H22 (B) and HepG2 (C) with different concentration. IC50 calculated from MTT data (D). Data is represented as mean ± SD (N = 3).

Cytotoxicity of D-SPB NPs *in vitro* for SH-SY5Y, H22 and HepG2 cells were evaluated based on MTT assay. There was no obvious cell cytotoxicity for blank SPB NPs against all of these tumor cells even at the highest tested concentration (**Figure S7**), suggesting a good cytocompatibility of blank SPB NPs. In sharp contrast, free DOX, D-SPB30, D-SPB50 and D-SPB150 displayed a dose-dependent cytotoxicity (**Figure 4A-C**). It was found that D-SPB NPs showed a lower cytotoxicity than free DOX under the same concentration. This can be ascribed to the slow DOX release from D-SPB NPs. Noticeably, D-SPB30 had a relatively higher cytotoxicity than D-SPB50 and D-SPB150 in the same cell lines at the same concentration. The IC50 value of D-SPB NPs was in order of D-SPB30 < D-SPB50 < D-SPB150 (**Figure 4D**). This is due to the larger cellular uptake for smaller D-SPB NPs, leading to higher cytotoxicity for D-SPB30. For the same D-SPB NPs but different cell lines, the IC50 value was in order of HepG2 cells < H22 cells < SH-SY5Y cells. The IC50 value of HepG2 cells was smaller than H22 cells and SH-SY5Y cells, indicating that the phenylboronic acid moiety which binds to SA overexpressed in tumor cells plays an important role in cytotoxicity of D-SPB NPs.

### Alleviation of tumor IFP and solid stress by SPB NPs

**Figure 5:**
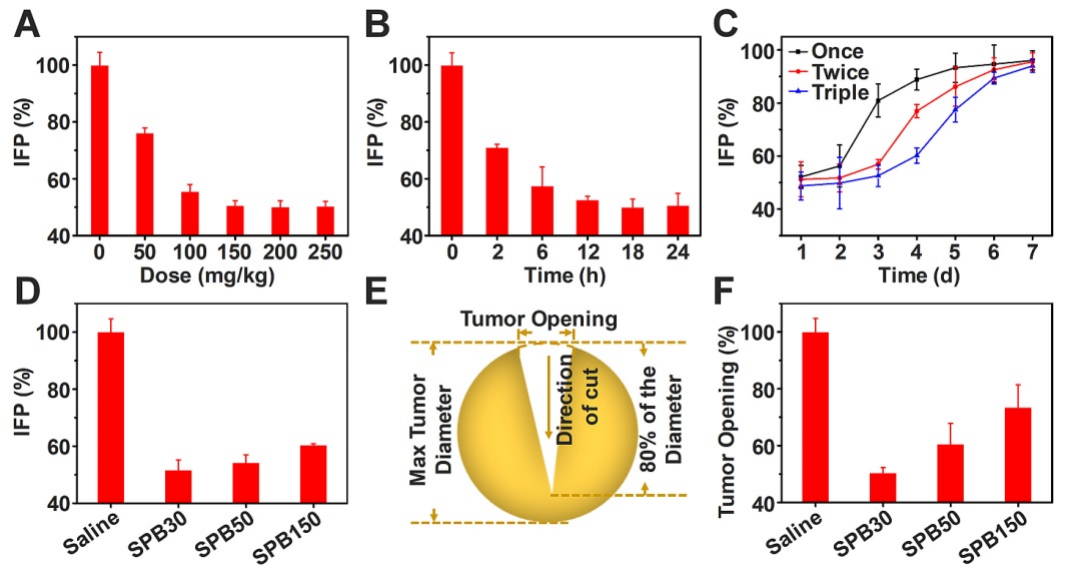
** Reduction of tumor IFP and solid stress.** Values of IFP treated with different doses of SPB30 at 24 h after i.v. injection (A); Values of IFP treated with SPB30 at a dose of 150 mg/kg at different times after i.v. injection (B); Time-dependent recovery of IFP after treatment with SPB30 by i.v. injection of once, twice and three times (C); Values of IFP treated with different sizes of SPB NPs at a dose of 150 mg/kg at 24 h after i.v. injection (D); Solid stress measurement diagram based on tumor opening after being treated with SPB NPs (E); Tumor opening at the surface of the tumors after being treated with SPB NPs for 24 h (F). Data is represented as mean ± SD (N = 3).

The elevated IFP and solid stress of tumor significantly hinder drug delivery and penetration into deep tumor site 9. Due to fast growth of tumor tissues, the stress was installed inside tumors. In contrast of IFP which is exerted by the fluid and compresses lymphatic vessels, solid stress is exerted by the nonfluid components of tumors and compresses blood vessels, leading to reduction of blood flow. Thus, reducing both IFP and solid stress of tumors can improve tumor microenvironment and enhance drug delivery in tumor. SP has received considerable attention for their great potential in decreasing risk of cardiovascular disease and lowering blood pressure 39. Whether SPB NPs have the ability to decrease tumor IFP and alleviate solid stress is worth investigating. To this end, 50 mg/kg, 100 mg/kg, 150 mg/kg, 200 mg/kg and 250 mg/kg of SPB30 were injected into H22 tumor-bearing mice through the tail vein. Tumor IFP after 24 h treatment was measured. As shown in** Figure 5**, the treatment of SPB30 significantly decreased tumor IFP which has not been reported yet. As the dose increased from 50 mg/kg to 150 mg/kg, the tumor IFP was decreased gradually from 76% of original value to 50% of one. However, the tumor IFP did not continually decrease when the injection dose of SPB30 increased from 150 mg/kg to 200 mg/kg, even 250 mg/kg, suggesting that 150 mg/kg SPB30 was an optimal dose to acquire a desirable tumor IFP reduction (**Figure 5A**). Meanwhile, the tumor IFP reduction with time was evaluated at a doge of 150 mg/kg SPB30. The tumor IFP decreased gradually to 50% of original value within first 18 h and then tended to be steady to 24 h (**Figure 5B**). Multiple injections of 150 mg/kg SPB30 programed with 24 h interval were able to stabilize the tumor IFP reduction for a long time. The double and triple injections slowed down the recovery rate of tumor IFP and triple injections provided a superior efficacy (**Figure 5C**). Finally, the effect of nanoparticle size on decreasing tumor IFP was investigated and all the three SPB NPs could decrease tumor IFP while SPB30 displayed the best efficacy with a decrease of 48% (**Figure 5D**).

Encouraged by tumor IFP reduction based on SPB NPs, the influence of SPB NPs on tumor solid stress was further investigated. A dose of 150 mg/kg of SPB NPs with different particle sizes was injected into CT26-bearing mice through tail vein, respectively. At 24 h after treatment, the mice were sacrificed humanely and tumors were excised. The collected tumors were cut through the middle to about 80% along the longest diameter and put in saline for 5 min (**Figure 5E**). After that, the tumor opening was measured and solid stress was calculated 35 as a function of particle size. A dose of 150 mg/kg of SPB NPs with different particle sizes was injected into CT26-bearing BALB/c mice through tail vein, respectively. As shown in **Figure 5F,** SPB30 was the most effective in reducing tumor solid stress among the three types of SPB NPs. SPB30 lowered tumor stress to about 50% of original value, which was 1.3 and 1.9 folds lower than SPB50 and SPB150, respectively. The great influence of SPB NPs on decreasing tumor IFP and solid stress was very obvious and should bring much benefit in drug delivery system. In order to explore the mechanism of SPB NPs on decreasing tumor IFP and solid stress, H22 tumors before and after treatment of SPB NPs were collected and cut into sections with a thickness of 20 μm. Collagen I, hyaluronan and TGF-β1 were chosen as three possible factors and stained by corresponding antibodies 10. The amount of these factors was observed by CLSM (Figure S8). There was no significant difference in the mean fluorescence intensity before and after treatment (Figure S9). Since the collagen I, hyaluronan and TGF-β1 didn't change significantly before and after administration of SPB NPs, we further suppose that it is associated with the ability of SP to inhibit angiotensin I-converting enzyme (ACE) 24, 25, which leads to dilating blood and lymphatic vessels of tumors.

### Tumor perfusion and penetration of SPB NPs *in vivo*

**Figure 6:**
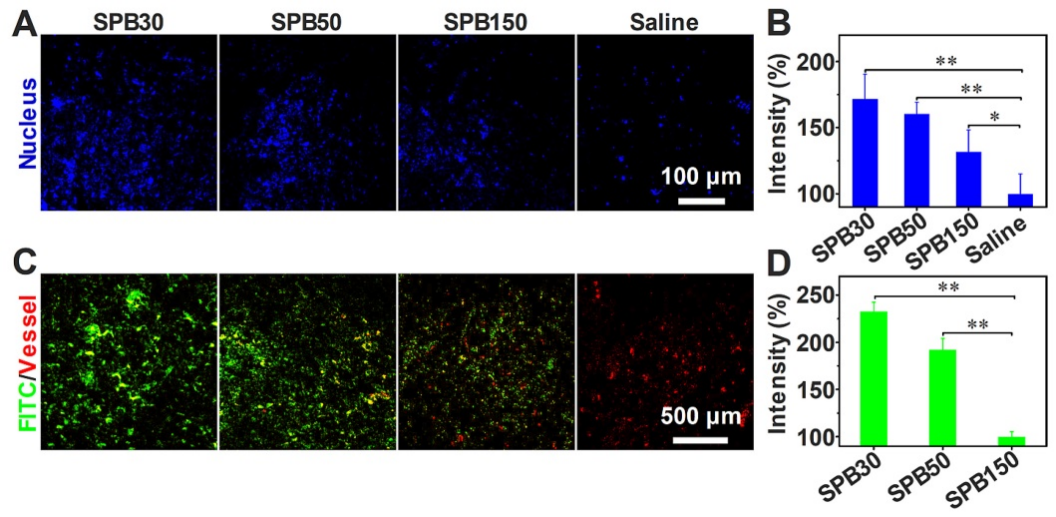
** Tumor perfusion and penetration of SPB NPs.** CLSM images of tumor blood perfusion after treatment with SPB NPs (A); The normalized average fluorescence intensity of Hoechst 33342 obtained from the corresponding CLSM images (B); Penetration behavior of FITC-labeled SPB NPs in H22 tumors at 24 h after i.v. injection, green stands for nanoparticles and red represents blood vessels (C); The normalized mean intensity of FITC obtained from the corresponding CLSM images (D). Data is represented as mean ± SD (N = 3). (* represents P < 0.05, ** represents P < 0.01).

To visualize the benefit after decreasing tumor IFP, tumor tissue perfusion was examined according to the method reported 40. After the i.v. injection of SPB NPs in the dose of 150 mg/kg, the aqueous solution of Hoechst 33342 was intravenously injected into H22 tumor-bearing mice to stain the cell nucleus of tumor tissues. When tumor IFP and solid stress decreased, more Hoechst 33342 should perfuse into tumor site and more cell nucleus should be stained. As seen in **Figure 6A,** a significantly higher blue fluorescence intensity was observed in tumor tissue treated with SPB NPs compared to that treated with saline, suggesting that the blood perfusion at tumor tissue was greatly improved after treated with SPB NPs. The mice treated with SPB30 showed the best perfusion in the tumor site among three sizes of SPB NPs (**Figure 6B**).

In order to evaluate the accumulation and penetration ability of SPB NPs *in vivo*, FITC-labeled SPB NPs were i.v. injected into H22 tumor-bearing mice. A significantly higher green fluorescence intensity was observed in tumor tissue of SPB30-treated mice compared to that of SPB50, SPB150 and saline-treated mice (**Figure 6C**). This indicated that SPB30 provided a largest accumulation in tumor tissue and penetrated farthest from tumor vessels (red). In contrast, SPB150 were localized around the blood vessels while SPB50 accumulated more in tumor site and penetrated deeper than SPB150. Such size-dependent penetration in tumor tissue *in vivo* suggests that SPB NPs with smaller size can accumulate more and penetrate further in tumor (**Figure 6D**).

### Free DOX biodistribution and antitumor efficiency after pretreatment

**Figure 7:**
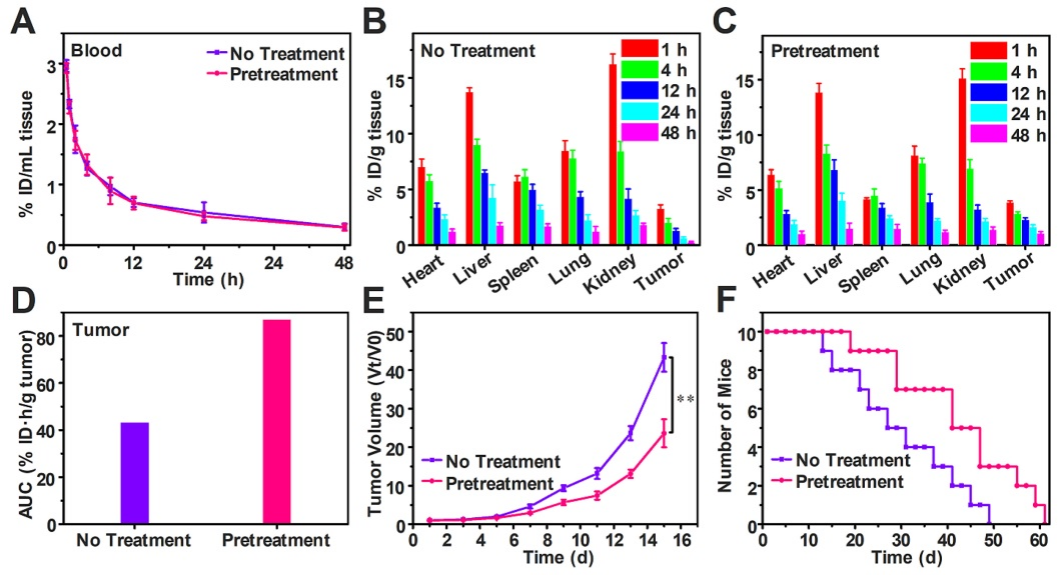
** Free DOX biodistribution and antitumor efficiency after decreasing tumor IFP and solid stress *in vivo*.** Time course of DOX concentration in the plasma of H22 tumor-bearing mice with and without pretreatment (A); Biodistribution of DOX in H22 tumor-bearing mice without pretreatment (B) and with pretreatment (C), data is represented as mean ± SD (N = 3); Accumulation of DOX in tumor calculated as area-under-the-curve (AUC) (D); *In vivo* tumor growth curves (E) and survival number (F) of H22 tumor-bearing mice with and without pretreatment, data is represented as mean ± SD (N = 10). (** represents P < 0.01).

To study the effect of decreasing tumor IFP and solid stress on biodistribution and antitumor efficiency of free DOX, H22-tumor bearing mice were intravenously injected with SPB30 for three days in the dose of 150 mg/kg each day before free DOX (4 mg/kg) i.v. injection. The DOX content in the blood and tissue samples were measured and calculated as the percentage of injection dose (% ID) per milliliter or gram. Compared to the mice without pretreatment, the half-life time of DOX in blood circulation, t_1/2_ was not changed after pretreatment (**Figure 7A**). However, DOX concentration in heart and spleen decreased after pretreatment. In particular, the DOX concentration of tumor in the pretreated group was higher than that of group without pretreatment at all time points (**Figure 7B and C**). The tumor area-under-the-curve (AUC) based on DOX concentration over time was used to compare the accumulation of DOX in tumor area. The tumor AUC of the pretreated group was 87.12% ID·h/g, which was 2.02 folds higher than the group without pretreatment. The significant increase in tumor AUC indicates that more DOX is accumulated in tumor after pretreatment with SPB30 (**Figure 7D**). Consequently, the antitumor efficiency of free DOX was changed before and after pretreatment although the antitumor agent was not changed. The antitumor efficiency of free DOX significantly improved after the pretreatment with SPB30, compared to the non-pretreatment group. The tumor volume of the non-pretreatment group increased faster and was 1.83 folds larger than the pretreated group at the fifteenth day (**Figure 7E**). The median survival time was 41 days for the mice pretreated and 27 days to the non-pretreatment group, indicating that decreasing tumor IFP and solid stress before administration of free DOX has a significant effect on inhibiting tumor growth and prolonging the survival time (**Figure 7F**).

### Biodistribution and antitumor efficiency of D-SPB NPs

**Figure 8:**
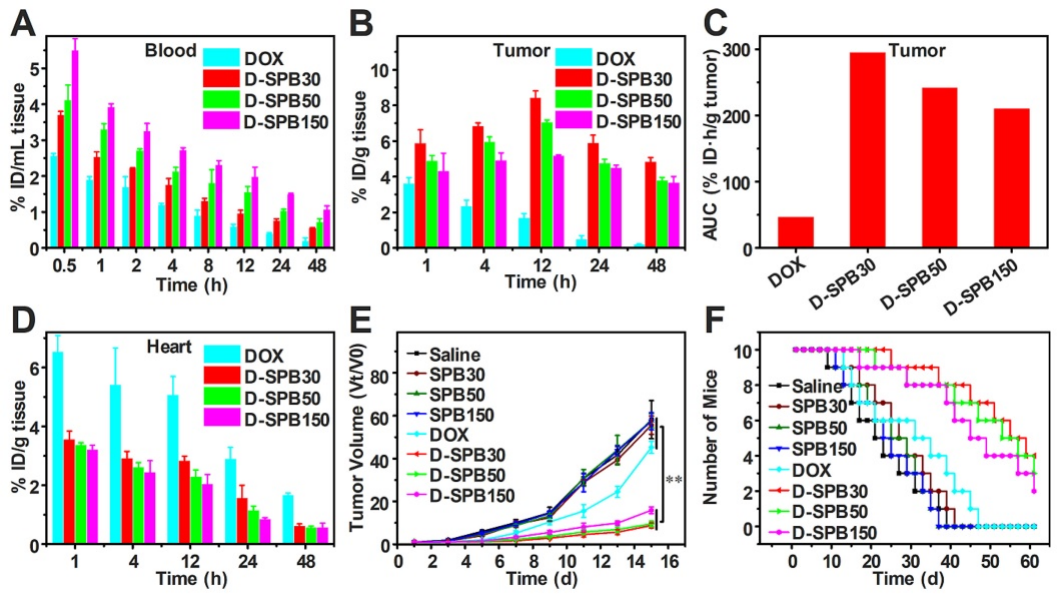
** Biodistribution and antitumor efficiency of D-SPB NPs.** Biodistribution of DOX in blood (A), tumor (B) and heart (D) of H22 tumor-bearing mice at various time points after i.v. injection; Accumulation of DOX in tumor was calculated as area-under-the-curve (AUC) (C), data is represented as mean ± SD (N = 3); *In vivo* tumor growth curves (E) and survival number (F) of H22 tumor-bearing mice after eight different treatments, data is represented as mean ± SD (N = 10). (** represents P < 0.01).

To estimate the drug delivery efficiency of soy protein nanoparticles themselves, biodistribution and antitumor effect of free DOX and D-SPB NPs with different sizes in H22 tumor-bearing ICR mice were carried out. Free DOX, D-SPB30, D-SPB50 and D-SPB150 were intravenously injected into the H22 tumor-bearing mice with a dosage of 4 mg/kg for DOX. It was noteworthy that the DOX concentration in blood for D-SPB30, D-SPB50 and D-SPB150 were 1.41, 1.59 and 2.06 folds higher than that of free DOX at 0.5 h (**Figure 8A**). The t_1/2_ of D-SPB30, D-SPB50 and D-SPB150 was 3.87 h, 6.33 h and 8.20 h, respectively. They were much longer than that of free DOX (1.84 h), suggesting that these SPB NPs can significantly prolong the circulation time of DOX in blood. In tumor tissue, DOX accumulation for SPB NPs showed a continuous increase with time. The maximum DOX accumulation in tumor for D-SPB30, D-SPB50 and D-SPB150 reached 8.44% ID/g, 7.06% ID/g and 5.18% ID/g at 12 h post-injection (**Figure 8B**). These values were much larger than that of free DOX formulation and are 4.92-fold, 4.12-fold and 3.03-fold higher than that of free DOX. Moreover, D-SPB NPs had a higher DOX accumulation in tumor than free DOX formulation at all the time. The D-SPB30 had the highest tumor AUC of 295.50 % ID·h/g tumor, which was 6.28 folds higher than free DOX and 1.22, 1.40 folds higher than D-SPB50, D-SPB150, respectively (**Figure 8C**). These results indicate a size-dependent accumulation of D-SPB NPs in tumor site. In addition, D-SPB NPs remarkably reduced DOX accumulation in heart (**Figure 8D**) and therefore might lower the cardiotoxicity of free DOX. Except in liver, the DOX concentrations in spleen, lung and kidney for all of these three sizes of SPB NPs were also lower than free DOX (**Figure S10**).

H22 tumor bearing mice were used to evaluate the antitumor efficiency of D-SPB NPs. DOX with a dose of 4 mg/kg or equivalent was intravenously injected into the mice when the tumor volume reached to about 100 mm^3^ on average and this day was defined as day 1. The tumor volume was determined using a Vernier caliper and the body weights were recorded every other day. It could be seen that the tumor volume treated with saline, SPB30, SPB50 and SPB150 increased fast, indicating that these four treatments have no effect on H22 tumor inhibition. The growth rate of tumor treated with free DOX was inhibited to some extent. In sharp contrast, the treatment of D-SPB30, D-SPB50 and D-SPB150 showed a much greater inhibition effect on tumor growth (**Figure 8E**). Among D-SPB NPs, the tumor volume of mice received treatments of D-SPB30 and D-SPB50 had statistical significance to D-SPB150 (**Figure S11A**). The TGI of free DOX, D-SPB30, D-SPB50 and D-SPB150 on day 15th was 22%, 84%, 83% and 73%, respectively. The survival curves of H22 tumor-bearing mice also supported these results. All of the mice in saline, SPB30, SPB50 and SPB150 groups died on the 41st day after treatment. Differently, there were two, eight, seven and six mice survival on the 41st day for the groups of free DOX, D-SPB30, D-SPB50 and D-SPB150, respectively (**Figure 8F**). The median survival time for the mice received different treatments was 21 (saline), 23 (SPB150), 25 (SPB50), 27 (SPB30), 31 (free DOX), 45 (D-SPB150), 53 (D-SPB50) and 55 (D-SPB30) days. A strong suppression of tumor growth and significant life elongation of mice bearing H22 tumor were achieved by the treatment of D-SPB NPs. On the other hand, the body weight of mice in all groups were slightly increased except free DOX group, suggesting the biosafety of SPB NPs (**Figure S11B**).

## Conclusions

Phenylboronic acid-decorated SPB NPs with diameters of 30 nm, 50 nm and 150 nm were prepared in a complete aqueous solution without any organic solvent. By virtue of the affinity between phenylboronic acid and SA and the intrinsic biological property of SP, such SPB NPs could not only actively target the SA overexpressed tumor model, but also decrease tumor IFP and solid stress, improving tumor microenvironment. The *in vivo* experiments demonstrated that pretreatment with SPB30 significantly increased the accumulation of free DOX in tumor, thus enhancing the antitumor efficiency of free DOX, owing to the reduction of tumor IFP and solid stress induced by pretreated with SPB30. Moreover, due to the size effect, the DOX-loaded SPB30 itself also showed much greater efficacy in accumulating in tumor tissue, impeding tumor growth and prolonging the lifetime of mice, compared to other groups.

Our study thus provides an effective nanoparticles design strategy to actively target and normalize tumor microenvironment for cancer therapy because the intrinsic biological property of SP let SPB NPs decrease tumor IFP and solid stress to enhance tumor penetration. In addition to chemotherapy drugs, other therapeutic drugs can also be integrated with such nanoparticles for other therapeutic approaches.

## Supplementary Material

Supplementary figures.

## Figures and Tables

**Table 1:** D_h_ and Zeta potential of SPB NPs.

Size	pH of Solution	ACVA (mg)	D_h_ (nm)	PDI	Zeta Potential (mV)
SPB30	6.2	10.0	30 ± 2	0.17	-12±3
SPB50	5.9	10.0	50 ± 4	0.14	-16±4
SPB150	5.5	10.0	150 ± 3	0.21	-23±4

**Table 2:** D_h_, DLC and DLE of D-SPB NPs.

Size	Diameter (nm)	PDI	DLC (%)	DLE (%)
D-SPB30	32±3	0.18	27.4	94.5
D-SPB50	53±6	0.19	27.6	95.2
D-SPB150	148±5	0.23	27.8	96.1

## Abbreviations

AC: acryloyl chloride
ACVA: 4,4'-Azobis(4-cyanovaleric acid)
ANOVA: Analysis of variance
APBA: N-3-acrylamidophenylboronic acid
AUC: area-under-the-curve
B16-F10: murine melanoma
BSA: bovine serum albumin
CLSM: confocal laser scanning microscopy
CT26: mouse colon carcinoma cell
DAPI: 4',6-diamidino-2-phenylindole
DLC: drug loading content
DLE: drug loading efficiency
DLS: dynamic light scattering
DMEM: Dulbecco's modified eagle medium
DOX: doxorubicin
D-SPB NPs: DOX-loaded soy protein nanoparticles
D-SPB30/D-SPB50/D-SPB150: DOX-loaded SPB30/ SPB50/SPB150
FBS: fetal bovine serum
FITC: fluorescein isothiocyanate
H22: murine hepatic tumor cell
HepG2: human liver tumor cell
i.v: intravenously
IC50: half maximal inhibitory concentrations
IFP: interstitial fluid pressure
MCTs: multicellular tumor spheroids
MTT: 3-(4,5-Dimethylthiazol-2-yl)-2,5-diphenyltetrazolium bromide
MWCO: molecular weight cut off
PBA: phenylboronic acid
PBS: phosphate buffer saline
RM6240: multi-channel physiological signal acquisition system
RPMI-1640: roswell park memorial institute
SA: sialic acid
SDS-PAGE: sodium dodecyl sulfate polyacrylamide gel electrophoresis
SH-SY5Y: human neuroblastoma tumor cell
SP: soy protein
SPB NPs: soy protein nanoparticles
SPB30/ SPB50/ SPB150: soy protein nanoparticles of 30/ 50/ 150 nm
TEM: transmission electron microscopy
TGI: tumor growth inhibition
TNF: tumor necrosis factor
% ID: percentage of injection dose
