# Supplementary material for: Targeting and microenvironment-improving of phenylboronic acid-decorated soy protein nanoparticles with different sizes to tumor
Source: Theranostics. 2019 Oct 11;9(24):7417–30. doi: 10.7150/thno.33470 (PMC6831295; doi:10.7150/thno.33470)
Supplement: Supplementary file 1 — Supplementary figures. [file thnov09p7417s1.pdf]

Supplementary Data

Targeting and microenvironment-improving of phenylboronic acid-decorated soybean protein nanoparticles with different sizes to tumor

Xiaoping Qian[1], Lei Ge[1], Kangjun Yuan[1], Cheng Li[1], Xu Zhen[1], Weibo Cai[2], Xiqun Jiang[1]✉

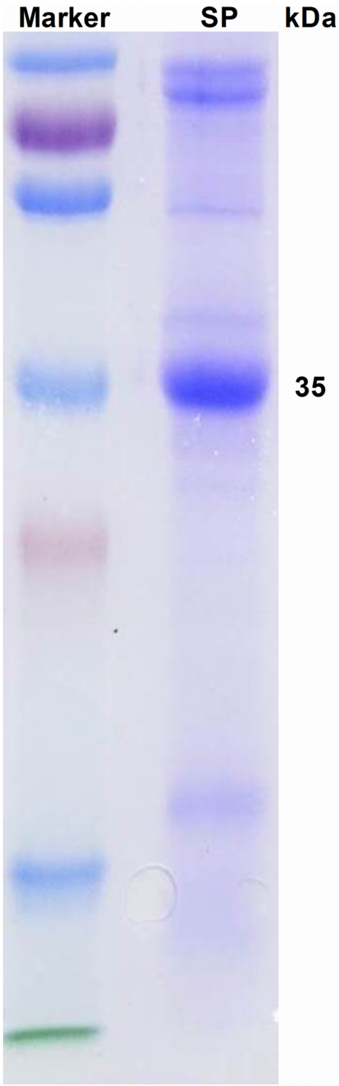

Figure S1. SDS-PAGE banding patterns of purified SP.

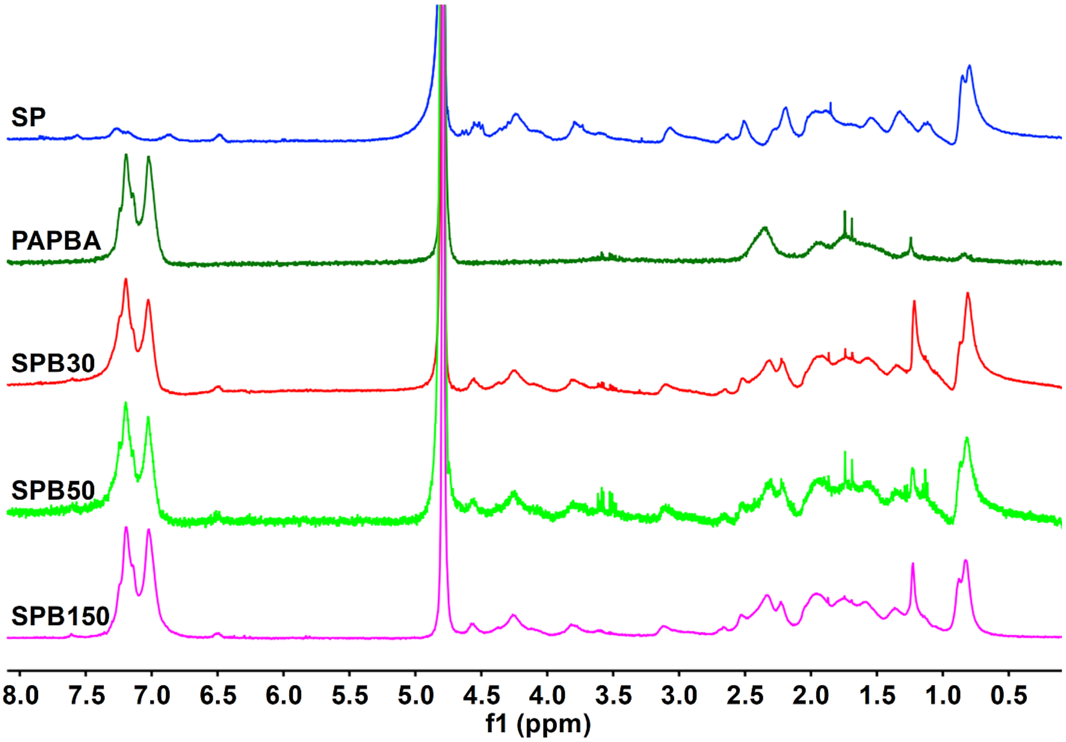

Figure S2. ^{1}H NMR spectra of SP, PAPBA, SPB30, SPB50 and SPB150 in D_2O with NaOD.

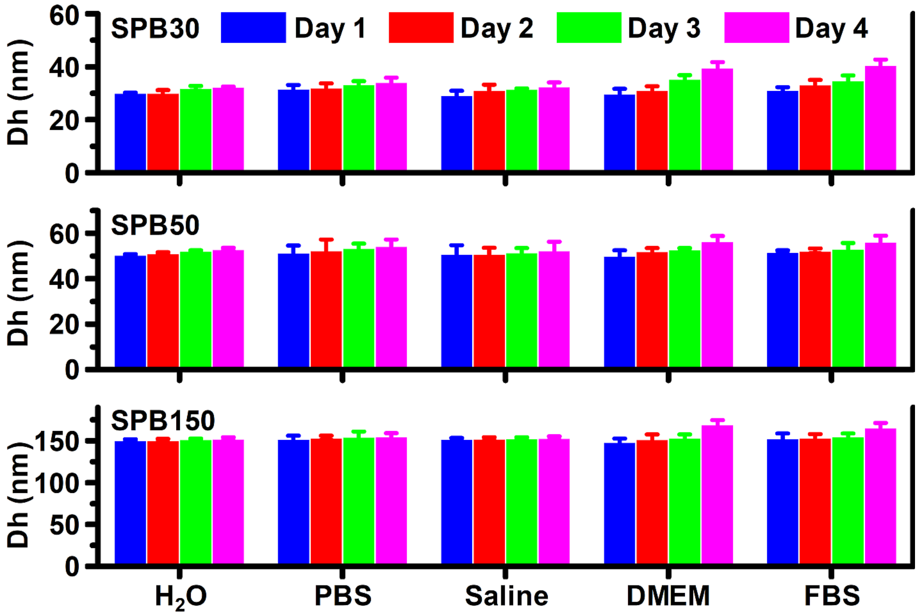

Figure S3. Stability of SPB30, SPB50 and SPB150 in distilled H_2O, PBS, saline, DMEM and 10% FBS solution. Data is represented as mean ± SD (N = 3).

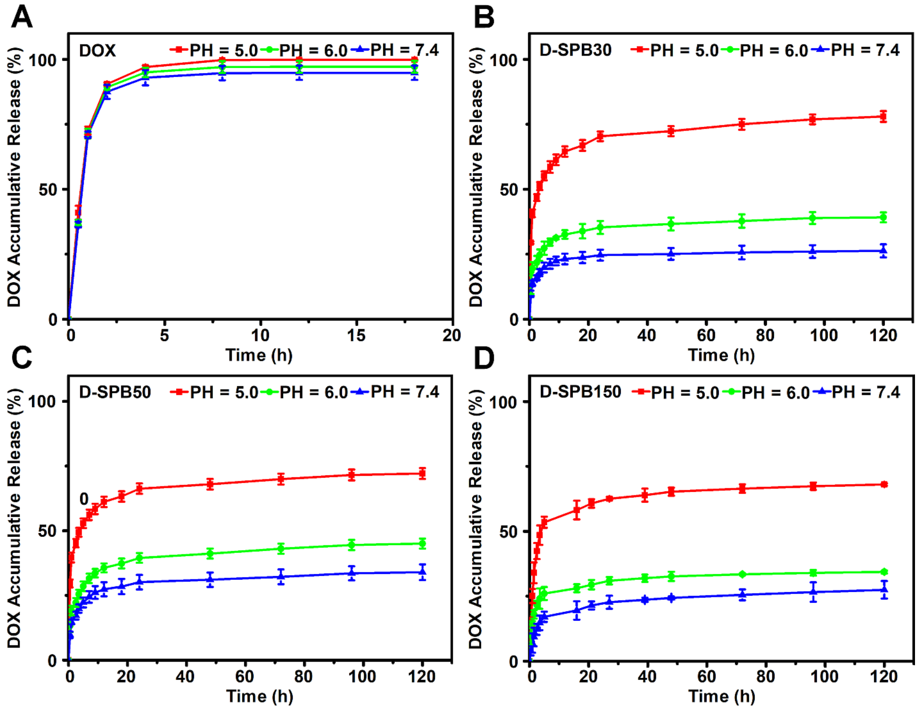

Figure S4. In vitro release profile of DOX from free DOX (A), D-SPB30 (B), D-SPB50 (C) and D-SPB150 (D) in 0.01 M PBS at PH 5.0, 6.0 and 7.4 at 37 ºC. Data is represented as mean ± SD (N = 3).

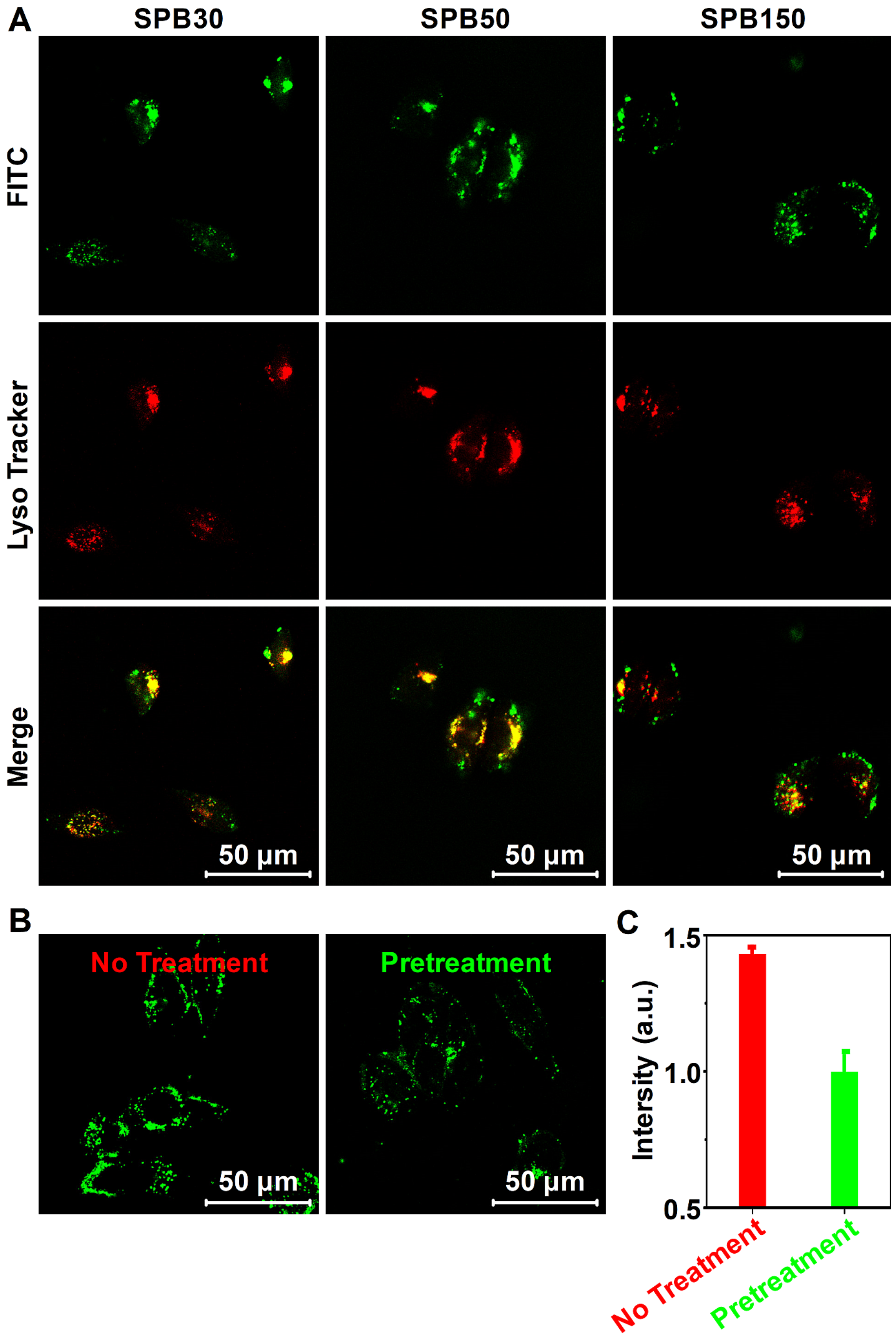

Figure S5. CLSM images of HepG2 cells after 4 h incubation with FITC-labeled SPB NPs at 37 ^{o}C. Endosomes/ lysosomes of the cells were marked by Lyso Tracker (red) (A). CLSM images of HepG2 cells after 4 h incubation with FITC-labeled SPB30 with or without pretreatment of APBA (B) and their quantitative data received by flow cytometry (C).

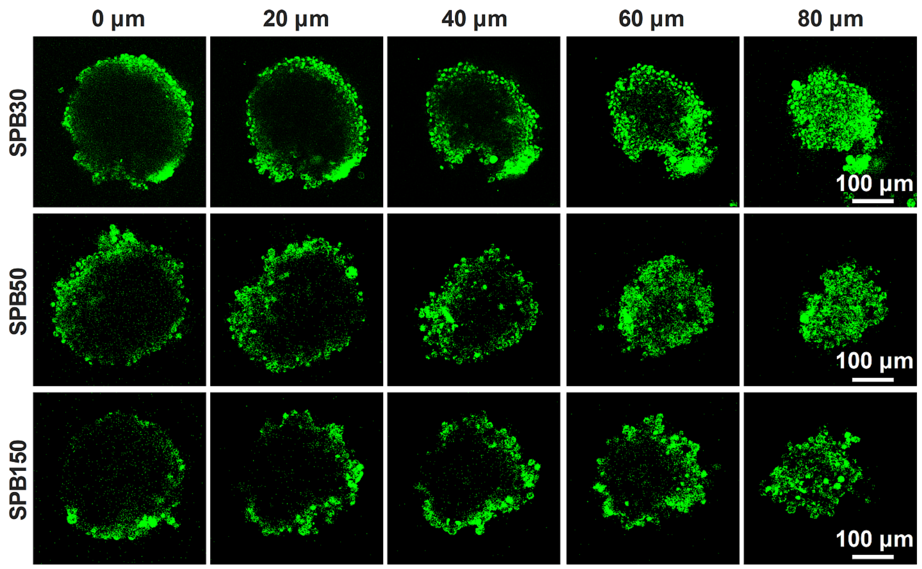

Figure S6. Representative Z-stack CLSM images of HepG2 MCTs incubated with FITC-labeled SPB NPs for 24 h were obtained starting in the middle of the spheroid in 20 μm intervals.

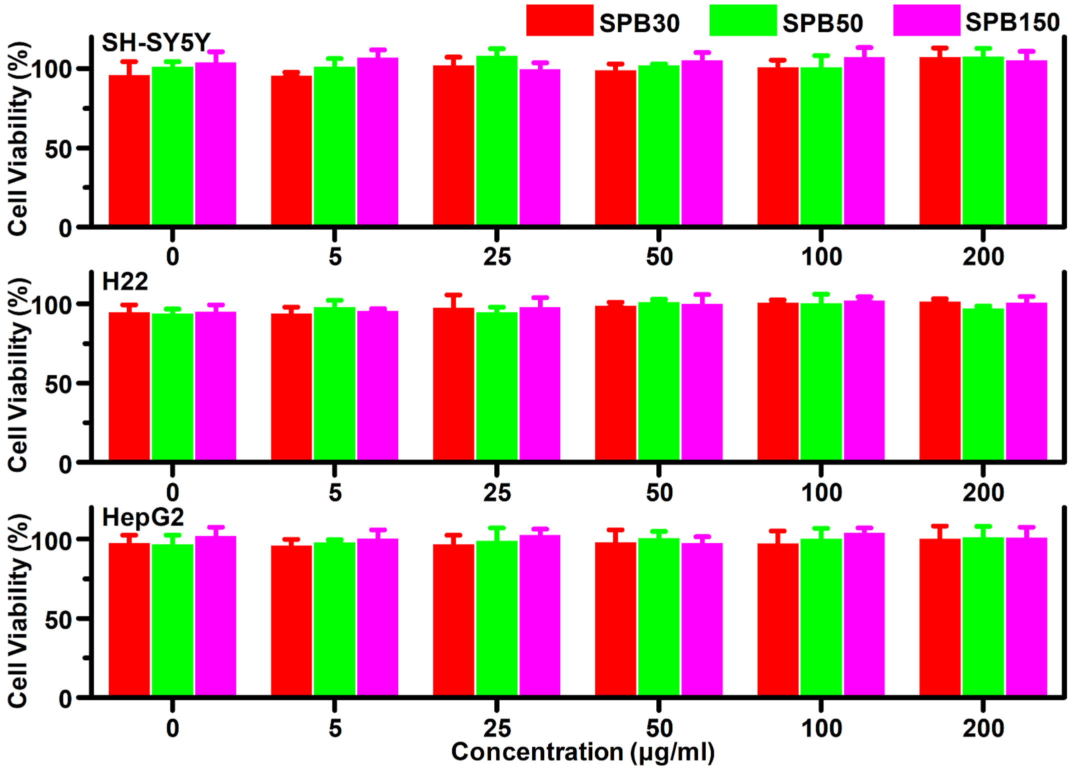

Figure S7. In vitro cytotoxicity of blank SPB NPs against SH-SY5Y, H22 and HepG2 cells. Data is represented as mean ± SD (N = 3).

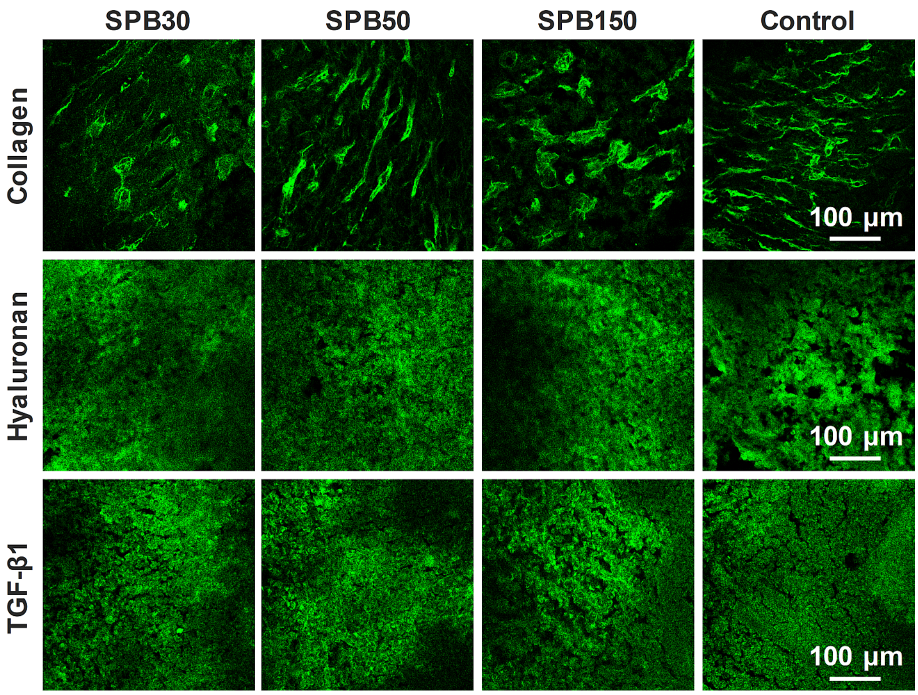

Figure S8. CLSM images of collagen I, hyaluronan and TGF-β1 of sliced H22 tumor before and after treatment with SPB NPs.

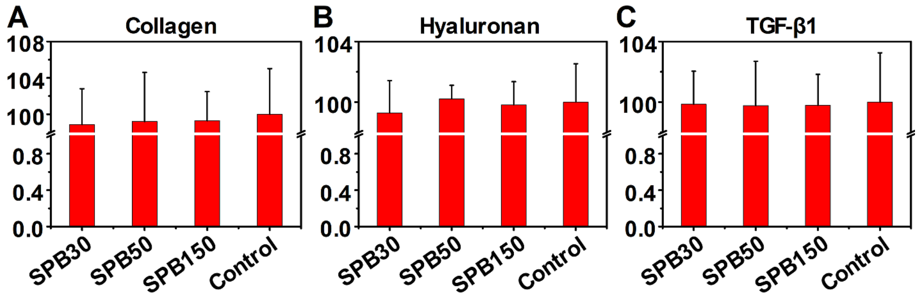

Figure S9. The normalized average fluorescence intensity of collagen Ⅰ, hyaluronan and TGF-β1 obtained from the corresponding CLSM images in Figure S8.

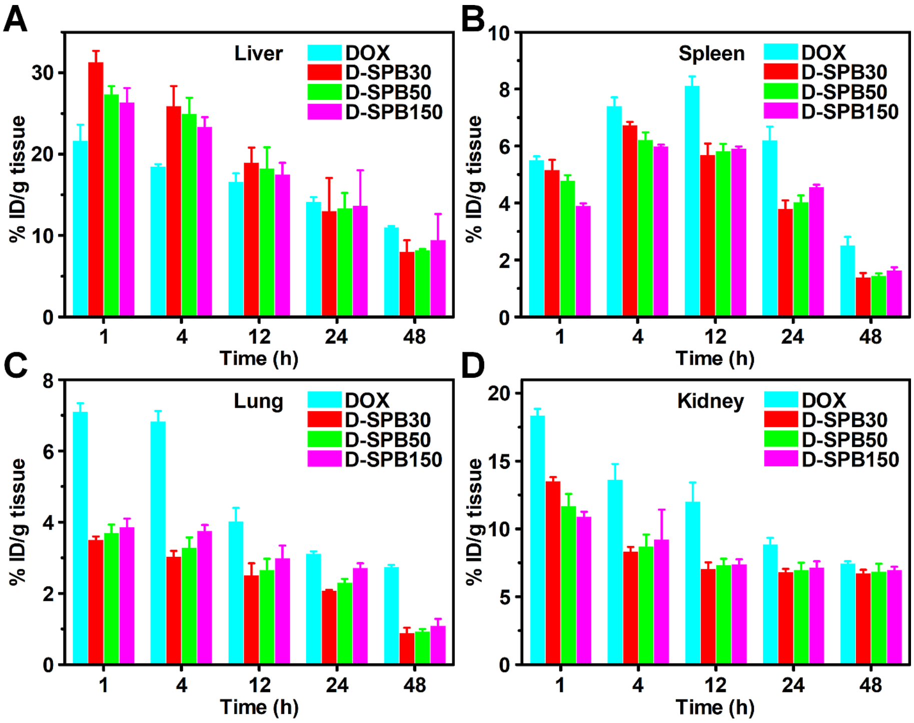

Figure S10. Biodistribution of DOX in liver (A), spleen (B), lung (C) and kidney (D) of H22 tumor-bearing mice at various time points after i.v. injection of D-SPB NPs. Data is represented as mean ± SD (N = 3).

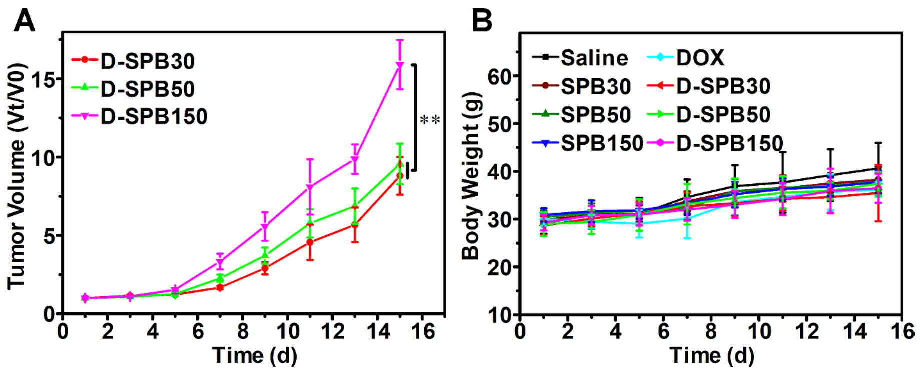

Figure S11. In vivo tumor growth curves (A) and body weight change (B) of H22 tumor-bearing mice. Data is represented as mean ± SD (N = 10). (** represents $P < 0.01$)
